# Recent Approaches to the Plasticization of Poly(lactic Acid) (PLA) (A Review)

**DOI:** 10.3390/polym16010087

**Published:** 2023-12-27

**Authors:** Elena E. Mastalygina, Kristine V. Aleksanyan

**Affiliations:** 1 Scientific Laboratory “Advanced Composite Materials and Technologies”, Plekhanov Russian University of Economics, 36 Stremyanny Ln., Moscow 117997, Russia; 2 Emanuel Institute of Biochemical Physics, Russian Academy of Sciences, 4 Kosygin St., Moscow 119991, Russia; 3 Engineering Center, Plekhanov Russian University of Economics, 36 Stremyanny Ln., Moscow 117997, Russia; 4 Semenov Federal Research Center for Chemical Physics, Russian Academy of Sciences, 4 Kosygin St, Moscow 119991, Russia

**Keywords:** poly(lactic acid) (PLA), plasticization, additives, elongation at break, glass transition point, polymer blends, copolymerization, reactive mixing

## Abstract

Poly(lactic acid) (PLA) is a polyester attracting growing interest every year in different application fields, such as packaging, cosmetics, food, medicine, etc. Despite its significant advantages, it has low elasticity that may hinder further development and a corresponding rise in volume of consumption. This review opens a discussion of basic approaches to PLA plasticization. These considerations include copolymerization and blending with flexible polymers, introducing oligomers and low-molecular additives, as well as structural modification. It was demonstrated that each approach has its advantages, such as simplicity and low cost, but with disadvantages, including complex processing and the need for additional reagents. According to the analysis of different approaches, it was concluded that the optimal option is the application of copolymers as the additives obtained via reactive mixing to PLA and its blends with other polymers.

## 1. Introduction

In recent decades, traditional large-scale polymers, which are products of petrochemical synthesis, are gradually being replaced by biodegradable polymers, the synthesis of which is carried out from plant raw materials, at least as packaging material, disposable tableware, and medical and hygiene products.

Aliphatic and aromatic polyesters of hydroxycarboxylic acids are characterized by not only biodegradability but also biocompatibility and inertness, which makes them suitable for use in medical and hygiene products [1]. Polyesters are characterized by a high ability for enzymatic hydrolysis including poly(lactic acid) (PLA), poly(3-hydroxybutyrate) (PHB) (class of poly(hydroxyalkanoates) (PHA), poly(ε-caprolactone) (PCL), poly(butylene succinate) (PBS), poly(butyleneadipate) (PBA), poly(butylene adipate terephthalate) (PBAT), poly(butylene succinateadipate) (PBSA). At the same time, PLA is the most durable, widespread and cheapest polyester from this series [2].

Poly(lactic acid)or poly(lactide) (PLA) is an aliphatic thermoplastic polyester with properties comparable to, for example, the widely used poly(ethylene terephthalate). PLA has a backbone formula of (C_3_H_4_O_2_)_n_ or [–C(CH_3_)HC(=O)O–]_n_, formally obtained by condensation of lactic acid C(CH_3_)(OH)HCOOH with loss of water. It can also be prepared by ring-opening polymerization of lactide [–C(CH_3_)HC(=O)O–], the cyclic dimer of the basic repeating unit [3].

**Figure 1:**
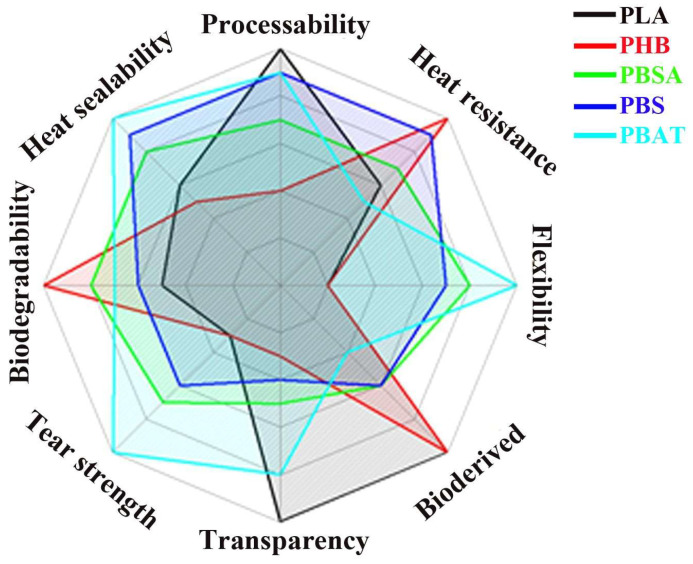
Comparison chart for biodegradable polyester properties (Reprinted from Compatibilization strategies and analysis of morphological features of poly(butylene adipate-co-terephthalate) (PBAT)/poly(lactic acid) PLA blends: A state-of-art review. Aversa, C.; Barletta, M.; Cappiello, G.; Gisario, A. Eur. Polym. J. 2022, 173, 111304. https://doi.org/10.1016/j.eurpolymj.2022.111304 with permission from Elsevier [5]).

PLA has become a popular material due to its economic production from renewable resources. In 2021, PLA had the highest consumption volume of any bioplastic in the world [4], although it is still not a commodity polymer. PLA is widely used in the following areas: medicine, the production of industrial and food packaging, the automotive industry and electronics [4]. Moreover, among all 3D printing materials, PLA is one of the most popular component for materials used for additive manufacturing for filament fabrication. The basic technology of additive manufacturing from PLA is fused deposition modeling (FDM). Figure 1 illustrates a comparison of the performance of PLA with that of other common biodegradable polyesters [5].

PLA is characterized by an increased elastic modulus; however, it has low impact strength [6], which makes it unsuitable for the manufacture of flexible polymeric products. The elongation at the break of PLA is 2.5–6% [7], which initiated the prospect of plasticizing it to increase flexibility.

It is also important to note that PLA is characterized by a narrow temperature-viscosity processing range and low melting strength, which causes additional difficulties when processing this polymer [8]. In addition, PLA is prone to hydrolytic degradation during processing at elevated temperatures in the presence of moisture absorbed from the environment. Polymer chains undergo thermal, thermomechanical and hydrolytic degradation due to high temperature and shear, which results in a significant loss in molecular weight.

It should be noted that PLA is biodegradable just at temperatures higher than its glass transition point (approximately 60 °C). The decomposition of PLA below its glass transition point occurs at extremely low rates (up to 5 years) [9]. Therefore, to utilize PLA, it is advisable to use the industrial composting method under conditions in which the temperature can spontaneously increase to 65–70 °C. Unfortunately, the industrial composting infrastructure is currently not well developed. Therefore, the development of approaches to regulating its decomposition period is an urgent task. Another problem is that PLA decomposes under composting conditions, releasing carbon dioxide and water, which increases its carbon footprint and reduces its environmental friendliness. Therefore, the related task of modifying PLA is to accelerate the process of biodegradation by using components capable of more accelerated degradation [10].

**Figure 2:**
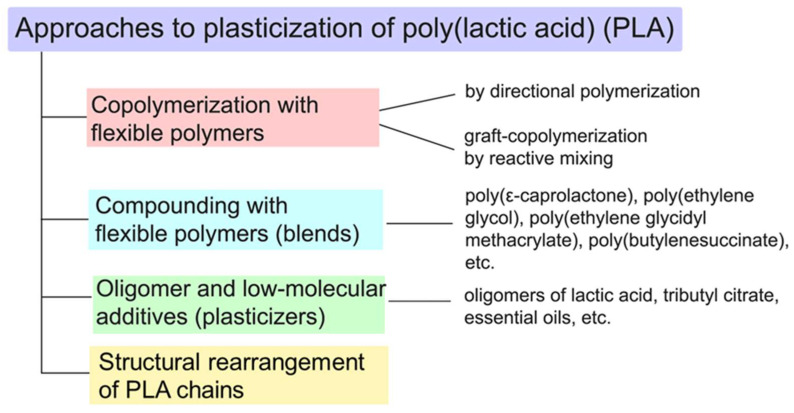
Basic approaches to the plasticization of PLA.

To regulate elasticity in PLA, the primary approaches are known: copolymerization with flexible polymers, compounding with flexible polymers (for example, PBAT, PBSA and modified natural rubber), use of low molecular weight and oligomeric plasticizers and the structural rearrangement of PLA chains [11,12,13]. This review provides an analysis and comparison of these primary approaches to plasticizing PLA (Figure 2).

The thermophysical properties of PLA are also an appropriate parameter for monitoring plasticization. Plasticization can be monitored by a decrease in the glass transition point and the degree of crystallinity of PLA, as well as the appearance of additional exothermic peaks of PLA recrystallization on heating thermograms (so-called cold crystallization) [14]. In [15], the effect of plasticization was also analyzed by reducing the dielectric and calorimetric glass transition temperatures and a systematic decrease in temperatures and enthalpies of crystallization and melting. The effects reflect a reduction in crystal nuclei, which is confirmed by optical microscopy, as fewer, albeit larger, crystals are formed in the copolymers.

The changes in the thermophysical properties are explained by an increase in the mobility of PLA macrochains in the presence of a plasticizer. Therefore, another indicator of plasticization is a rise in the molecular mobility of the polymer (segmental and local) [16]. It is precisely due to the increase in the flexibility of the system that a structuring effect on PLA crystallites is observed. Hernández et al. [17] assessed the effect of plasticization by changing the rheological properties of the melt. They found that changing the chemical structure of the PLA block copolymer (PLLA-b-polyether-b-PLLA) causes changes in the glass transition and melting points, which significantly affect the rheological properties.

The integral parameters for determining plasticization are the physical and mechanical characteristics of PLA. The determining parameter for the effectiveness of the plasticization is an increase in tensile elongation and impact strength [18,19]. Moreover, a change in these indicators can be associated both with an increase in segmental mobility in the amorphous regions of the polymer and with the influence of modifiers on the supramolecular structure.

## 2. Copolymerization with Flexible Polymers

An effective method for modifying PLA is through the creation of its copolymers. Copolymerization with more flexible polymers makes PLA elastic. In some cases, creating such copolymers allow one to regulate the physicomechanical characteristics and increase thermal stability, improve the rheological properties of the melt and impart greater biodegradability. It is important to note that, thanks to copolymerization, it is possible to create materials based on incompatible polymers when mixing results in weak interfacial interaction [20]. To obtain copolymers of PLA with other copolymers, two main approaches are widely used: directional polymerization with the production of copolymers of a given composition and structure and graft-copolymerization by reactive mixing (as a rule, extrusion) in a melt in the presence of an initiator and/or catalyst. Reactive extrusion is a fast and cost-effective method commonly used for the functionalization of PLA or ring-opening polymerization (ROP) of lactide (for example, [21,22]).

**Figure 3:**
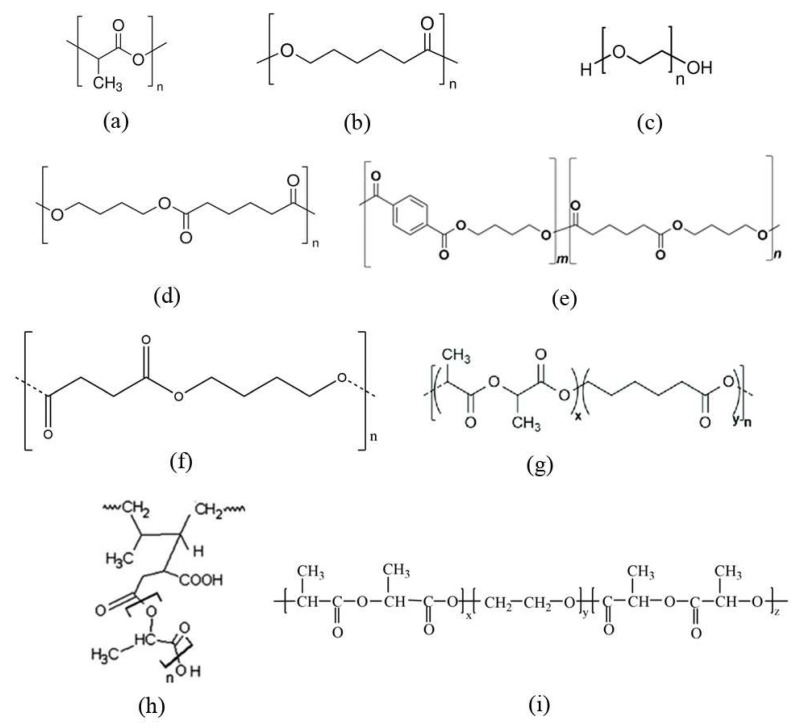
Structural formulas of PLA (**a**), PCL (**b**), poly(ethylene glycol) (PEG) (**c**), PBA (**d**), PBAT (**e**), PBS (**f**), block copolymer of poly(L-lactide-co-ε-caprolactone) (PLA-PCL) (**g**) [27], graft-copolymer of poly(lactic acid)-g-natural rubber (PLA-g-NR) (**h**) [18] and triblock copolymer of poly(D-lactic acid-co-ethylene glycol-co-D-lactic acid) (PDLA-PEG-PDLA) (**i**) [28].

PCL, a linear semi-crystalline polyester, is often used to improve the properties of PLA (structural formulas of the monomer unit are in Figure 3). In addition to the plasticization effect, an increase in biodegradability in the natural environment can be achieved due to the lower crystallinity and greater susceptibility to hydrolysis of PCL. A long-chain branched block copolymer (LB-PCLA) of PLA and PCL was synthesized in [23]. When comparing the synthesized copolymers with pure PLA and PLA/PCL blends, it was shown that elongation at breakup increased 30-fold. In addition, the copolymers had more pronounced shear-thinning and longer relaxation times, which can be explained by their long-chain branched structure. Similar patterns were found for (AB)_n_ multiblock copolymers based on PLA and PCL obtained by the chain extension reaction with hexamethylene diisocyanate [24], and random copolymers of poly(L-lactide-co-ε-caprolactone) (PLLCL) (50/50 wt%) [25,26,27]. The copolymers demonstrated increased elongation at breakup to 800%. An increase in the thermal decomposition temperature compared to pure PLA was also shown, which is an advantage for the industrial process. In all studies, no noticeable deterioration in tensile strength was observed.

Along with directed copolymerization, the technique of reactive mixing of PLA with other polymers is used to create PLA copolymers. For example, a copolymer with a ratio of 95:5 was obtained by reactive mixing of PLA with poly(ethylene glycidyl methacrylate) (EGMA) in the presence of a DCP initiator [29]. The PLA/EGMA copolymer had an elongation at a break 58 times higher than that of pure PLA while maintaining high tensile strength and transparency.

Natural rubber (NR) can be used as a plasticizing additive in PLA [30,31]. Despite the ability of NR to exert a physical plasticization effect on PLA, when creating PLA/NR mixtures, NR is distributed in the PLA matrix in the form of dispersed particles that demonstrate poor adhesion to PLA. There are known works on the creation of graft-copolymers poly(lactic acid)-g-natural rubber (PLA-g-NR) [18] by grafting malleated natural rubber (MNR) onto low-molecular weight PLA. Two types of copolymers were prepared to have anhydride moieties of 10 and 20 wt%, the addition of which to PLA resulted in a 200% improvement in impact strength and a two-fold percentage rise in elongation at break. Based on these results, it was also found that PLA-g-NR can improve the properties of PLA to a greater extent than NR due to its higher compatibility.

Numerous studies have shown the effectiveness of using poly(ethylene glycol) (PEG) to create highly flexible PLA copolymers. PEG is a polyether compound and has exceptional elasticity. Among various plasticizers and impact modifiers, PEG is one of the most effective due to its wide range of molecular weights, non-toxicity, miscibility and biodegradability (for example, [32]). PEG plasticization can effectively increase the chain mobility of PLA as well as improve its ductility and stretchability, thereby expanding the range of potential applications. Using biodegradable block-copolymers with both hydrophilic and relatively hydrophobic functions allows one to create flexible, partially hydrated and biocompatible materials for medicine.

The creation of PLA-PEG copolymers gives the possibility of controlling the properties of PLA more effectively. Directionally synthesized via ring-opening polymerization, poly(l-lactide)-b-polyethylene glycol-b-poly(l-lactide) (PLLA-PEG-PLLA) in the presence of an extender chain exhibited greater flexibility than PLLA thanks to the flexibility of the PEG middle blocks [33]. Baimark et al. [34] and Wu et al. [28] showed similar patterns for PLLA-b-poly(ethylene glycol)-b-PLLA (PLLA-PEG-PLLA) block copolymers synthesized by polymerization of PLA with ring-opening and chain extender based on epoxy resin. In his work, Wu synthesized triblock copolymers of PEG (block B) type ABA in combination with PLLA (blocks A) with different molecular weights of PEG and different degrees of polymerization of PLLA [35]. The works showed the positive effect of the chain extender on the plasticization of PLA, so PLLA-PEG-PLLA samples with an extended chain did not have phase separation. As the length of the PEG blocks increased, the elasticity of the materials rose, the hardness decreased, and water absorption increased.

In another work, the graft-copolymerization of low-molecular-weight poly(ethylene glycol) acrylate (PEGA) to PLA through reaction mixing was successfully carried out. The presence of acrylated PEG grafted onto PLA resulted in a decrease in the glass transition point by more than 20 °C and an increase in elongation at the breakup from 4.7% to 17.9% [36].

PBS is another important biodegradable thermoplastic polyester available in the market. There are known works on the creation of graft-copolymers of PLA with PBS using reactive mixing in the presence of oligo(butylenesuccinate) and an initiator [19]. The introduction of flexible PBS chains reduced the glass transition point, leading to a decrease in hardness and elastic modulus. It is important to note that it is proposed to use PLA-co-PBS copolymers as individual polymers and as additives that improve compatibility in these PLA-PBS blends, which leads to decreased interfacial tension and improved impact strength and elongation.

In order to impart increased plasticity to PLA, polyesters containing flexible adipic acid units, such as PBSA [14] and poly(propylene adipate) [15,37], are widely used. As a rule, the synthesis is carried out by the ring-opening polymerization of lactides in the presence of oligomers of the modifying component, resulting in block-copolymer formation. It was shown that the introduction of flexible adipic acid units successfully increased the elongation and the rate of PLA biodegradation.

## 3. Compounding with Flexible Polymers (Blends)

When creating blends of the above polymers, there is a problem with their thermodynamic incompatibility. Thus, one of the tasks of researchers is to establish optimal ratios of polymers in terms of solubility parameters, as well as increase compatibility through the use of compatibilizers. The blending of two incompatible blends with varied melting temperatures is an additional factor promoting delamination of the blend. When cooled from the melt (in the case of melt compounding), the crystallization of each of the polymers occurs in its own temperature range. With a significant difference in temperature ranges, separation at the interface between the phases of blends and the formation of shrinkage cavities can occur [38]. It is important to note that the PLA and flexible polyesters used as modifying additives crystallize in different temperature ranges, which also promotes the delamination of the compositions.

Numerous studies have shown that the change in entropy when mixing high-molecular compounds is negligible; therefore polymer blends are thermodynamically incompatible, so the interaction of components is limited by interphase boundaries. However, creating polymer blends is widely practiced as the easiest way to create new materials. Even if there is no synergy compared to additive values, the blend combines the properties of both components. As a result of segmental solubility or mutual diffusion of segments of incompatible polymers, an interfacial layer is formed. Segmental solubility manifests itself in macroscopically uniform physical properties, primarily determined by the structure of the interfacial layer. Therefore, it is preferable to create the maximum possible phase interface, that is, reducing the size of the domains of the dispersed phase with increasing the surface area of the domains per unit volume of the blend.

Compounding flexible polyesters with PLA allows one to obtain materials with complementary properties, namely, increased elastic modulus and rigidity on the one hand, as well as flexibility and crack resistance; on the other, bypassing the expensive synthesis stage [39]. Among the most popular polymer additives, PCL, PBAT and PBSA also belong to the class of biodegradable polyesters, with physical and mechanical properties similar to thermoplastic elastomers [2]. The mixing with PBATis also affects the thermal characteristics, namely, the glass transition point of the blends decreases with the rise in the PBAT content [39]. The presence of adipic acid units in copolymers of PBAT and PBSA determines the high flexibility of the polymers and their increased ability to hydrolyze under the action of microbiological enzymes [40]. PCL has excellent macrochain flexibility, and its mechanical behavior is similar to rubbers.

Using the theoretical calculations based on the Flory–Huggins model, it is possible to evaluate the possibility of intermolecular interaction of polymers in the melt, which, in turn, determines the structure of the interphase layer, primarily its density and defectiveness [41]. In general, polymers are more compatible if they are of a similar nature and have similar Hildebrant solubility parameters. The higher the molecular weights of the blended polymers, the lower the predicted probability of the blend miscibility. Another tendency is that the higher the temperature, the higher the probability of the blend’s miscibility.

The study described in [42] provided a theoretical prediction of the miscibility for PBAT/PLA blends. It has been shown that a higher affinity would be possible between the two polymers when the BA and BT monomers reach a molar ratio exceeding 1 to 1. A pair of PLA and poly(ethylene oxide) (PEO), according to the Flory–Huggins interaction parameter, are miscible at nearly all temperatures [43]. The interaction parameters calculated from the Flory–Huggins equation in [44] for the blend of PLA with PBS showed a thermodynamically acceptable immiscible level. Relatively low levels of PBS addition improved the melt flow characteristics of the blend.

An important parameter when compounding polymers is the close values of melt viscosity. In most cases, at processing temperatures of 170–200 °C, the melt viscosity of flexible polymers (PCL, PBAT and PBSA) is lower than the melt viscosity of PLA. Therefore, to create systems with the maximum possible phase interface areas, it is preferable to use PLA as the dominant (larger) phase. For example, it was found in [45] that for PLA/PBSA mixtures, the size of PLA domains in a sample with 30 wt% PLA was larger than that of PBSA domains in a sample with 70 wt% PLA since a PBSA melt of lower viscosity is not capable of deforming (dispersing) the PLA phase. It was found that with a content of 30 wt% PBSA in the PLA matrix, the blend is characterized by a structure with a maximum specific interfacial surface area of PBSA domains, providing the maximum plasticizing effect on PLA.

The improvement of mechanical properties is considered a determining parameter for the effectiveness of plasticization. Flexible polyesters such as PCL, PBSA and PBAT have low glass transition temperatures, so at room temperature, their macromolecules are able to move freely, exhibiting flexibility [46]. The elongation at the breakup parameters for samples based on these polymers comprises 400–600% [47]. Therefore, the addition of PCL, PBSA or PBAT to PLA has a positive effect on its rigidity and brittleness. In addition, when flexible PBAT and PBSA are introduced into the structure of rigid PLA, the impact strength increases, which reduces the brittleness of such materials [48].

Sun et al. [49] showed an increase in the proportion and perfection of PLA crystallites in the presence of small PCL additives. An increase in impact strength parameters was shown with the introduction of small PBSA additives (10–20 wt%) [50] in PLA compared to parameter additive values, which reduces the fragility of materials. With a higher content of the plasticizing polymer in the blend, a negative deviation from the additive parameters was observed. At the same time, Lascano et al. [46] found that for PLA/PBSA blends, the optimal ratio was 70/30, which corresponds to increased relative elongation and impact strength. In [51], it was found that the elongation at break for PLA/PBAT mixtures increases to 600% with PBAT content of 15 wt%. A negative aspect of adding flexible PCL, PBAT and PBSA to PLA is the deterioration of optical properties, namely a decrease in the transparency of the latter [49].

To increase the miscibility of PLA and plasticizing polyesters, compatibilizers are used. Thanks to compatibilizers, chemical interactions and cross-links are formed at the polymer-polymer interface, which makes the material less defective and more homogeneous. It should be noted that compatibilizers do not affect the mutual solubility of polymers. The role of these additives is reduced to increasing the “connectivity” of the polymers, increasing the bond strength in the interfacial layer and, as a result, increasing the mechanical homogeneity of the mixture as a whole.

Triblock and diblock copolymers from blocks of compoundable polymers, epoxy-styrene-acrylic oligomers [46], as well as low molecular weight tributyl citrate [49,52], glycidyl methacrylate (GMA), dicumyl peroxide, triphenyl phosphite, and lysine triisocyanateare used as compatibilizers. Such compatibilizers can enhance the effect of the plasticizer polymer. There is also the practice of using PLA copolymers with grafted compatibilizers. For example, the study described in [53] describes the compositions of PLA/PBAT/PLA-g-GMA = 70/30/(1–10) wt%. When the compatibilizer PLA-g-GMA was more than 8 wt%, the elongation at breakup increased more than 200%. Adding poly(L-lactide-block-carbonate copolymers (PLA-b-PC) to 80PLA/20PCL blends reduced the PCL droplet size. At the same time, the glass transition temperature of the PLA phase is depressed, as the PLA-b-PC addition induces partial miscibility between PLA and PCL [54].

**Figure 4:**
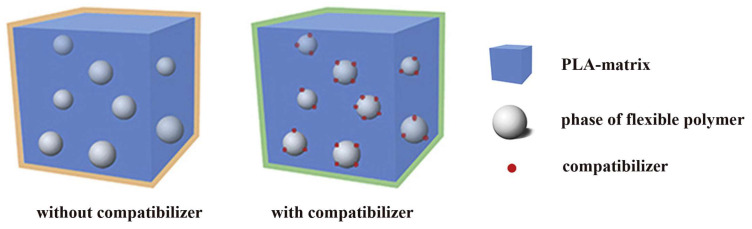
Schematic diagram of PLA blended with flexible polymers with and without the use of a compatibilizer.

Figure 4 schematically shows the architecture of PLA blends with flexible polymers without/with the use of a compatibilizer. Since the flexible polymer content is usually no more than 30 wt%, the materials have a structure in the form of a PLA matrix with domains dispersed inside it (dispersed phase) of the flexible polymer. If present, the compatibilizer concentrates at the interface. The size and degree of dispersion of domains depend not only on the ratio of polymers but also on the technology for obtaining the materials and the presence of compatibilizer.

The biodegradability of aliphatic polyesters is caused by the hydrolytic cleavage of chains at the ester bonds of aliphatic units under the action of water and microbial enzymes. It is important to note that PLA, unlike PCL, PBAT and PBSA, is subjected to rapid hydrolysis only at elevated temperatures, which limits the conditions for its composting [55]. The addition of easily hydrolyzable flexible polyesters will provide a higher rate of PLA hydrolysis.

The possibility of regulating the diffusion and barrier properties by varying the ratio of PLA and PBAT in mixtures [41,56], as well as the type and content of plasticizer [57], has been proven. Thus, it is possible to create materials with different rates of release of active additives by changing the diffusion parameters of a polymer matrix based on PLA/PBAT or PLA/PBSA in different ratios.

Blending PLA with flexible polymers is a common plasticization method to produce commercial products. It is widely known that a large-scale biodegradable polymer material produced by Novamont S.p.A. (Novara, Italy) under the Mater-Bi brand and its recipe is constantly undergoing changes. The patent for Novamont S.p.A. (CA 2662105 from 2008) [58] describes a biodegradable multiphase composition containing a continuous phase of a hydrophobic polymer (poly(esters) or poly(amides)), a dispersed phase of starch in the form of nanoparticles with sizes less than 0.3 μm and an additional dispersed phase of a hard and brittle polymer with an elastic modulus of more than 1000 MPa (PHA, PLA or poly(glycolic acid) (PGA)).

The US patent 8747971 from 2014 by BASF describes the general composition of the material based on 25–60 wt% of starch, 40–75 wt% of synthetic aliphatic-aromatic copolyester of the commercial product Ecoflex from BASF (Ludwigshafen, Germany), 6–15 wt% of glycerol [59]. The following polymers and their blends were used in different years as synthetic aliphatic-aromatic copolyesters, which are the main carrier matrix of the material: PLA, PCL, PBA, PBAT, and others. For example, a material under the Ecovio brand by BASF consists of the compostable BASF polymer Ecoflex (61–67 wt%), PLA (Ecopla, by Cargill, Neuss, Germany) (7–13 wt%), and other additives. The invention ensures the production of thin films by blown extrusion with an elastic modulus of more than 300 MPa.

## 4. Oligomers and Low-Molecular Weightadditives (Plasticizers)

The introduction of oligomeric and low-molecular-weight plasticizers effectively improve the mobility of the PLA matrix chains. Thanks to the additional application of plasticizers, one can achieve optimal technological properties and flexibility of the materials. In particular, it is expanding the temperature range of the PLA processing.

As mentioned above, thanks to its specific characteristics, PEG is well known and often used as a plasticizer for PLA, especially its oligomers [13,60,61,62,63,64,65,66,67]. The calculations of theoretical compatibility made in the paper [68] suggest that the PLA and PEG blends are likely miscible at low PEG concentrations (10–30 wt%), but they become apparently immiscible at higher PEG content (>50 wt%).

The comparison of the effect of the PEG molecular weight on the material characteristics showed that the lower molecular weight PEG promoted the improvement in the compatibility and ductility of PLA to a significant degree [61,69]. It should be noted that the introduction of PEG improves plasticity and affects biodegradability since it is a water-soluble substance. With regard to its thermal properties, it was established that the strongest decrease in glass transition point was for the lowest molecular weight PEG. However, the mechanical characteristics deteriorated in time due to the PEG migration (sweating out). This effect can be avoided by the introduction of some substances, such as carbon black, calcium carbonate, zeolite, bran and chitin/cellulose fibrils (for example, [70,71,72] etc.). However, the attempt to stop migration is leading to deterioration of the plasticity. There are works devoted to the use of PEG not only as a plasticizer for PLA but also as a technological additive improving adhesion between the layers in the case of producing products using the FDM technique. The heat treatment during FDM influences the penetration of PEG between the PLA chains and decreases the intermolecular forces along the chain, making the PLA more flexible [73]. An alternative to PEG is polypropylene glycol (PPG) [74].

Among the most studied low-molecular plasticizers for PLA are derivatives of hydrocarboxylic acids; first of all, esters, for example, of citric acid, since they are nontoxic and miscible with PLA. However, some studies consider esters as little-explored acids, namely tartaric acid [75], cinnamic acid [76], etc. Tributyl citrate [77,78,79], as well as acetyl tributyl citrate [57,70,78,80,81,82], have demonstrated a good effect on elongation at the breakup parameter—increasing the plasticizer content led to a rise in ductility. Harte et al. [78] showed that the rise in plasticizer content resulted in a decrease in the glass transition point; there was no low-temperature antiplasticization effect. An amount of 10 wt% content of plasticizers led to a decrease in elastic modulus and tensile strength; the further rise in the content of plasticizers deteriorated the mechanical characteristics. This can also be connected with the migration of plasticizers at high concentrations in the composites.

However, Jing et al. [83] compared two plasticizers for PLA, namely glycerol and tributyl citrate. The presence of the single glass transition point for the latter additive, in contrast to two glass transition points in the case of glycerol, indicates that tributyl citrate is more compatible with PLA. With the use of mesoscopic dynamic simulations, it was confirmed that there is a phase separation between PLA and glycerol.

The comparative study of adipate esters, citrate esters and glycerol for plasticization of PLA-based materials was performed in [84]. The most efficient plasticization was achieved with adipate esters, thanks to their linear structure. The elongation at breakup for the PLA-based material with adipate esters is higher than for tributyl citrate (both ranging from 0.7 to 1.0%): 126–148 and 72–103%, respectively. When glycerol was used, it was impossible even to form the pellets since the material was not homogeneous. When cooled, it became brittle and sticky due to excess of glycerol. It is not a suitable plasticizer for PLA, but it is good for starch. That is the reason why there are numerous works dedicated to PLA-based materials plasticized by thermoplastic starch with the same low-molecular weight plasticizers, glycerol, adipate etc. [84,85,86,87,88]. Müller et al. [87] plasticized PLA with thermoplastic starch containing 36 and 47 wt% of glycerol. It was demonstrated that glycerol partitioned into two phases: starch and PLA. The major part of glycerol was in thermoplastic starch, while it did not diffuse into the PLA. The authors used thermodynamic modeling and predicted some dissolution of PLA in starch but not vice versa. It was concluded that starch led to significant deterioration of the PLA properties.

In addition, the oligomers of lactic acid are used to plasticize PLA. It was demonstrated that such plasticizers can be effective up to 25 wt% content (elongation at break up to 300%) [89]: there was a significant decrease in the glass transition point. The authors noted that no phase separation took place, and the material ductility increased. However, with higher plasticizer content, the components are immiscible. Avolio Roberto et al. [90] added two oligomers of lactic acid, carboxyl or hydroxyl, end-capped as plasticizers. It was found that only 20 and 25% content of plasticizers resulted in ductile behavior, with the rise in elongation at breakup at 480%. Thus, the application of the oligomers of lactic acid as a potential plasticizer was proven.

One more interesting and gaining rising attention plasticizers for PLA are essential oils, such as sunflower [91,92,93,94,95], linseed [96,97,98,99,100], cottonseed [101,102], soybean [103,104,105,106,107,108,109,110], castor [111,112] oils, etc.

Wadhi and Weliam [95] demonstrated the application of epoxidized sunflower oil as a plasticizer for PLA using the solution casting method. Varying the component ratio, it was established that a 20% content of oil led to maximal values of elongation at breakup along with elevated thermal stability. Analysis of SEM micrographs proved that epoxidized sunflower oil is miscible with PLA. In turn, an innovative approach is realized in the utilization of fried sunflower oil (as a byproduct of fast food) as a plasticizer for starch to partially replace glycerol and mixed with PLA [109]. The application of maleinized linseed oil for plasticization of PLA with almond shell flour [104] and hazelnut shell flour [96] allowed one to improve the ductility of the materials, even at relatively low contents of oil.

Aydın et al. [106] demonstrated the application of the PLA-based systems obtained by solvent casting with modified soybean oil in medicine. As was shown above, the maximal elongation at breakup was observed at 20% oil content. Dai et al. [103] revealed that the epoxidized soybean oil accumulated mainly on the microcrystalline cellulose particle surface, and the elongation was improved to 38.5%. As a promising plasticizer and synthesized at first, epoxidized Brazil nut oil was used to improve the PLA ductility [113]. Even at 2.5 wt% of oil, one observed an improvement in the PLA brittleness. The maximal 70% elongation at breakup was achieved at 7.5 wt% of oil.

Another compound used to improve the mechanical properties of PLA is clay and its modifications (for example, [114,115,116,117,118,119,120,121,122,123,124]). Scaffaro et al. [122] used modified montmorillonite to improve the characteristics of the PLA-based material. It resulted in a rise in elongation at breakup at 300% and acceleration of biodegradability. In [120], the elongation at breakup was achieved at 43% with a 5 wt% content of montmorillonite. Shayan et al. [115] obtained a full green PLA-based nanocomposite with modified starch and reinforced with montmorillonite. Varying the component ratio, the ductility of the material was increased.

Thus, the wide range of low molecular-weight plasticizers can be used to change the mechanical and thermal properties and biodegradability. These plasticizers are characterized by low cost, accessibility, etc. However, they have some disadvantages, such as low temperatures for processing, a strong tendency to phase separate from the polymer matrix, migration and so on [90].

## 5. Structural Rearrangement of PLA Chains

**Figure 5:**
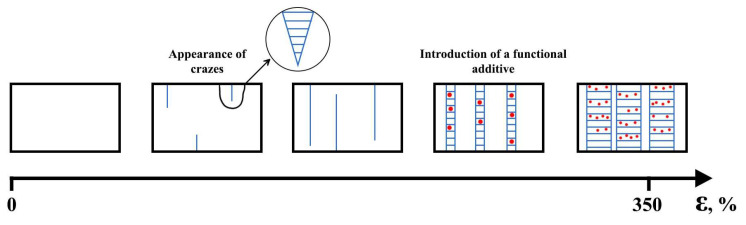
Scheme of the structural modification of a polymer with simultaneous impregnation of an additive.

In the case of glassy and partially crystalline polymers, crazing is often one of the main failure mechanisms [125,126,127]. However, an application of adsorption-active media can facilitate significantly regulating the process of crazing [128]. Structural-mechanical modification by the mechanism of crazing in an adsorption-active medium is a process of structural rearrangement of PLA during drawing with a formation of fibrillar structure. As a result, the formation of a fibrillar structure occurs, which is a system of oriented and separated fibrils with a diameter of 5–20 nm in the polymer volume [129]. At the same time, the capture, dispersion and fixation of a substance dissolved in the medium in the pores of the polymer matrix can occur. Thus, this method allows for the plasticization of PLA but also its functionalization. Figure 5 presents a schematic illustration of the structural modification of a polymer with simultaneous impregnation of an additive.

Trofimchuk et al. have carried out a lot of investigations in the field of exploration of PLA crazing [130,131,132,133]. In particular, the effect of different media (air and adsorption-active liquids) during uniaxial stretching on the formation of crazes in PLA was studied in [131]. The crazing mechanism was investigated in hydrocarbons, organosilicon liquids, aliphatic alcohols, and water-alcohol solutions. The maximal elongation at breakup was detected in n-pentanol (570%). Thanks to this investigation, one can predict the critical mechanical stress value when the crazing is initiated in PLA during application. In [134,135], the authors developed the functional fibrous material of PLA for application in medical practice using the crazing mechanism.

Zhou et al. [136] investigated PLA with low crystallinity (only 3%), which was subjected to physical aging at room temperature. The authors used post-stretching that improved not only the toughness of PLA but also increased the aging resistance. Investigation of the physical aging of PLA (observation of changes in properties of the injection molded samples in time) showed that large internal stresses led to crazing and, as a result, to brittle failure [137]. With the aim of understanding the plastic deformation mechanism of PLA upon cold-drawing, the authors in [138] used SAXS and AFM. It was revealed that crazing mechanisms are responsible for the brittle character of PLA. 

Stoclet et al., using an in situ SAXS method, illustrated that there are complex interactions between shear banding and crazing in PLA during plastic deformation. Billimoria et al. [139] also used SAXS and some other methods to investigate the mechanical and crystalline structure development during the uniaxial deformation of PLLA while varying strain rates and draw temperatures. As a result of thermal analysis, it was revealed that the drawing occurred due to the orientation of PLA molecular chains. It was shown that the rise in draw temperature resulted in crazing, microvoiding and cavitation. The authors concluded that regulating the parameters (temperature, rate) of the processes above can allow one to control and modify the PLLA-based material properties. Xu et al. [140] demonstrated that specific processing of PLA, namely extrusion casting under melt-stretching flow and cast roll temperature filed, resulted in a rise in elongation at breakup at 270%. The PLA molecular chains could be fully extended by varying the melt–draw ratios. In order to initiate the crazing resulting in improvement of ductility and toughness, the authors [141] mixed PLA with poly(1,4-cis-isoprene), a major component of natural rubber. It should be noted that three sequential mechanisms (including crazing) described in the work resulted in sufficiently efficient toughening of PLA at a small amount of poly(1,4-cis-isoprene) (5%). Other works also explore the changes in structure correlated with stresses, drawing etc. (for example [142,143,144,145]).

Mihai et al. [146] compared the changes in structure degradation during extrusion foaming using CO_2_ of pure PLA and PLA with thermoplastic starch. It was revealed that high CO_2_ concentration led to the plasticization of PLA, thereby increasing the chain mobility, which resulted in the acceleration of the crystallization.

An investigation of the surface of PLA with atactic PHB via SEM showed that crazing was an active deformation mechanism in all samples independently of the PHB content [147].

Despite the possibility of changing the characteristics of PLA and PLA-based materials using structural deformation techniques, it is admissible only as the final stage of the material processing.

## 6. Conclusions

The review demonstrates the ways to regulate plasticity and some associated characteristics (such as thermal and thermophysical parameters, rheological behavior, biodegradability, etc.) of PLA-based materials (Table 1). The basic approaches to improve PLA brittleness include copolymerization and blending with flexible polymers, introducing oligomers and low-molecular additives and structural modification. Each approach may provide good plasticity for PLA-based materials, but the application of any of them can be dictated by the final application field, further processing, etc. It can be concluded that an optimal is the application of copolymers obtained via reactive mixing as the additives to PLA and its blends with other polymers. Directed copolymerization of PLA with flexible polymers is also effective but is limited by low product yield and high cost. The use of PLA copolymers is justified not as individual materials but as compatibilizers for mechanical blends of PLA and the corresponding polymer. The introduction of oligomeric and low-molecular plasticizers is the simplest way to modify PLA; however, the effect of such plasticization persists for a short time due to the leaching of additives from the polymer. Among low-molecular additives, essential oils are of greatest interest since they provide not only plasticization but also enhancement in functionality (for instance, antimicrobial activity). Despite the high values of elongation at the breakup of PLA obtained via structural modification, the use of this approach is hindered by the fact that it must be the final stage of the material processing.

## Figures and Tables

**Table 1:** Analysis of the mechanical characteristics and *T_g_* of PLA-based materials obtained using various approaches to plasticization (n.d.—non-determined).

Approach	Tensile Strength (MPa)	Elongation at Breakup (%)	Impact Strength (kJ/m^2^)	*T_g_* (°C)	Ref.
Poly(L-lactide)-b-poly(ethylene glycol)-b-poly(L-lactide)	31	157	n.d.	30	[33]
Multiblock copolymer of poly(L-lactide-co-ε-caprolactone) (80/20 (*w*/*w*)) (reactive mixing with hexamethylene diisocyanate)	12	57	n.d.	n.d.	[24]
Poly(L-lactide-co--ethylene-glycidyl methacrylate) (95/5 (*w*/*w*)) (reactive mixing with dicumyl peroxide)	67	270	6	66	[29]
Poly(L-lactide) and block copolymer of poly(L-lactide) and poly(caprolactone) (85/15 (*w*/*w*))	47	210	n.d.	61	[23]
Poly(lactic acid) and poly(butylene adipate-co-terephthalate) blend (85/15 (*w*/*w*))	24	485	n.d.	61	[39]
Poly(lactic acid) and poly(butylene succinate adipate) blend (80/20 (*w*/*w*))	47	25	5	59	[50]
Poly(L-lactide) and poly(ethylene glycol) 400 (90/10 (*w*/*w*))	32.5	140	n.d.	50	[61]
Poly(L-lactide) and L-lactic acid oligomer (75/25 (*w*/*w*))	n.d.	300	n.d.	31	[89]
Poly(lactic acid) with epoxidized sunflower oil (80/20 (*w*/*w*))	18	230	n.d.	n.d.	[95]
Poly(lactic acid) (Bioflex (BF), trade name Bio-Flex^®^ F2110) + carvacrol + montmorillonite (90/5/5 (*w*/*w*/*w*))	8	300	n.d.	n.d.	[122]
Poly(lactic acid) in *n*-pentanol (AR grade) (uniaxial stretching)	12.5	570	n.d.	n.d.	[131]
Pre-oriented poly(lactic acid) (uniaxial stretching)	n.d.	270	n.d.	62	[140]

## Data Availability

Not applicable.
